# Adoptive transfer of IL13Rα2-specific T cells for the treatment of glioblastoma: building on clinical achievements with second-generation CARs

**DOI:** 10.1186/2051-1426-1-S1-P2

**Published:** 2013-11-07

**Authors:** Christine E Brown, Renate Starr, Araceli Naranjo, Brenda Aguilar, Wen-Chung Chang, Julie Ostberg, Charles Warden, Yate-Ching Yuan, Massimo D'Apuzzo, Michael E Barish, Behnam Badie, Michael C Jensen, Stephen J Forman

**Affiliations:** 1 Beckman Research Institute and City of Hope National Medical Center, Duarte, CA, USA; 2 Seattle Children's Research Institute, Seattle, WA, USA

## 

The prognosis of patients with high-grade glioma remains grim despite improvements in standard therapies including surgery, radiation and chemotherapy. Immunotherapy approaches targeting brain tumors offer promise over conventional treatments by harnessing the potency and specificity of the immune system. We have developed a chimeric antigen receptor (CAR) T cell immunotherapy for malignant glioma targeting IL-13 receptor α2 (IL13Rα2), a cell surface receptor over-expressed by a high percentage of high-grade gliomas, but not expressed at significant levels on normal brain tissue. Bioinformatic studies demonstrated that expression of IL13Rα2 is associated with increased glioma malignancy grade, mesenchymal signature gene expression, and reduced patient survival (top ~1% of single genes associated with GBM survival). Previous pre-clinical efforts employing 1st-generation CAR T cell clones comprised of a membrane-tethered IL-13 ligand and the intracellular CD3-zeta endodomain (termed IL13-zetakine) demonstrated potent MHC-independent IL13Rα2 anti-glioma activity against both bulk glioma populations, and glioma stem-like tumor propagating cells. Orthotopic glioma mouse models, however, suggest that multiple infusions of IL13-zetakine+ T cells are required for anti-tumor efficacy since T cell persistence is limited for these 1st-generation CAR T clones. Importantly, completion of two FDA-authorized Phase I clinical trials, evaluating both autologous and allogeneic IL13-zetakine T cell clones in 9 patients, demonstrates the feasibility of this approach, absence of serious side-effects, and provide evidence for transient anti-glioma tumor responses for patients with IL13Rα2pos glioblastomas. We now aim to enhance therapeutic efficacy of this approach by augmenting T cell effector function through the expression of CARs with co-stimulatory signaling in T cell population capable of long-term persistence. Indeed, we find that a single administration of central memory T cells engineered to express a 2nd-generation IL13-41BB-zetakine CAR can eradicate established glioma tumors in mice, thus outperforming the 1st-generation IL13-zetakine+ T cells. These findings support the potential for treating patients with high-grade glioma with IL13Rα2-specific CAR-engineered T cells.

